# Quantitative Detection of *Schistosoma japonicum* Cercariae in Water by Real-Time PCR

**DOI:** 10.1371/journal.pntd.0000337

**Published:** 2008-11-18

**Authors:** Yuen Wai Hung, Justin Remais

**Affiliations:** Center for Occupational and Environmental Health, School of Public Health, University of California, Berkeley, California, United States of America; Imperial College Faculty of Medicine, United Kingdom

## Abstract

In China alone, an estimated 30 million people are at risk of schistosomiasis, caused by the *Schistosoma japonicum* parasite. Disease has re-emerged in several regions that had previously attained transmission control, reinforcing the need for active surveillance. The environmental stage of the parasite is known to exhibit high spatial and temporal variability, and current detection techniques rely on a sentinel mouse method which has serious limitations in obtaining data in both time and space. Here we describe a real-time PCR assay to quantitatively detect *S. japonicum* cercariae in laboratory samples and in natural water that has been spiked with known numbers of *S. japonicum*. Multiple primers were designed and assessed, and the best performing set, along with a TaqMan probe, was used to quantify *S. japonicum*. The resulting assay was selective, with no amplification detected for *Schistosoma mansoni*, *Schistosoma haematobium*, avian schistosomes nor organisms present in non-endemic surface water samples. Repeated samples containing various concentrations of *S. japonicum* cercariae showed that the real-time PCR method had a strong linear correlation (R^2^ = 0.921) with light microscopy counts, and the detection limit was below the DNA equivalent of half of one cercaria. Various cercarial concentrations spiked in 1 liter of natural water followed by a filtration process produced positive detection from 93% of samples analyzed. The real-time PCR method performed well quantifying the relative concentrations of various spiked samples, although the absolute concentration estimates exhibited high variance across replicated samples. Overall, the method has the potential to be applied to environmental water samples to produce a rapid, reliable assay for cercarial location in endemic areas.

## Author Summary

Schistosomiasis ranks second only to malaria among parasitic diseases with regard to the number of people infected and those at risk. *Schistosoma japonicum* is the species that causes human and animal disease in China, the Philippines, and to a lesser extent, Indonesia. Recent evidence of schistosomiasis re-emergence in China has reinforced the need for active disease surveillance in these areas. Schistosomiasis infection occurs through contact with water contaminated with *S. japonicum* cercariae, the free-living stage of the parasite shed from intermediate host snails. Current practice of detecting cercariae in the environment uses sentinel mice, a method with serious limitations in which mice are exposed to environmental water and then maintained for 6 weeks before being dissected to count worms. The method is labor intensive and costly in terms of time and resources, making it logistically prohibitive to monitor water contact sites regularly or comprehensively. Here we develop a quantitative PCR assay to measure *S. japonicum* cercariae concentration in water, providing a potential method for rapid and reliable data collection in the field, potentially replacing the use of live animal models.

## Introduction

Recent evidence of schistosomiasis re-emergence has been reported in the hilly and mountainous regions of Sichuan Province, reinforcing the need for active disease surveillance in these areas [1]. Meanwhile, endemic areas with active transmission persist in China, with the most recent data indicating approximately 700,000 current infections. Importantly, in these areas with active transmission, infection rates have increased over the last decade [2]. This contrasts with the significant gains that had been made reducing the prevalence of schistosomiasis in China over the past five decades, down from an estimated 11.6 million people infected at the launch of the national control program in the 1950s [3]. Evidence of re-emergence, and increased transmission in endemic areas, suggest that nationwide elimination will require increased vigilance. Meanwhile, a recent global estimate suggests 207 million people are infected with schistosome parasites, with 779 million people at risk [4]. The species *Schistosoma japonicum* is responsible for intestinal and hepatosplenic schistosomiasis in humans and animals in China, the Philippines, and Indonesia [5], and has been associated with anaemia [6] and liver and colon cancers [7], among other outcomes. Current tools for environmental detection of the parasite are extremely limited.

Human infection occurs through contact with a free swimming larval stage of the parasite called cercariae. In western China where disease re-emergence has been reported, cercariae are released from infected snails inhabiting irrigation ditches supplying agricultural fields. In this environment, cercarial density is known to be spatially and temporally variable, and thus disease surveillance, along with preventative actions and risk assessments, would benefit from measurements of cercarial density in natural waters [8]–[10]. Indeed, attempting to estimate exposure-response relationships in the absence of quantitative data on the distribution and density of cercariae in the environment can lead to insufficient power to detect a response [10]. Yet current methods to quantify cercarial density in natural waters are seriously inadequate. The traditional technique involves suspending sentinel mice in cages just below the water surface, exposing them for five hours per day for two days. After exposure, the mice are returned to the laboratory, maintained for six weeks to allow for maturation of the parasite, then dissected to count any resulting worms *in vivo* [10]. The method is labor intensive and costly in terms of time and resources, making it logistically prohibitive to monitor water contact sites regularly or comprehensively, and raises ethical issues arising from the use of live animals for routine disease surveillance.

Molecular assays such as polymerase chain reaction (PCR) have shown promise for the detection of parasites in a variety of media. Qualitative techniques have been developed to detect the presence or absence of *S. japonicum* and avian schistosome cercariae recovered from snail hosts [11],[12], as well as *Schistosoma mansoni* cercariae in water [13]. Meanwhile, real-time PCR assays have been reported for the quantification of *S. japonicum* and other human schistosome eggs in human and animal stool [14]–[16], and for the quantification of *S. mansoni* DNA [17]. To date, no quantitative assay has been developed to detect *S. japonicum* cercariae in water samples. The objective of this study is to develop a fast, reliable TaqMan real-time PCR assay to detect and quantify *S. japonicum* cercariae in field samples, with the goal of improving surveillance capabilities, as well developing better estimates of environmental risk.

## Materials and Methods

### Schistosomes

Schistosome-infected snails were obtained from the Biomedical Research Institute funded by National Institute of Allergy and Infectious Disease (NIAID). The institute provided live *Oncomelania hupensis* (subspecies *hupensis*) snails exposed to the Chinese strains of *S. japonicum*, *Biomphalaria glabrata* snails exposed to the Puerto Rican strain of *S. mansoni*, and *Bulinus truncatus* (subspecies *truncatus*) snails exposed to the Egyptian strain of *Schistosoma haematobium*. All living snails were maintained for a period of 1 day to 3 weeks in facilities at the University of California, Berkeley. *O. hupensis* snails were crushed on a glass slide to maximize the extraction of live *S. japonicum* cercariae. *B. glabrata* snails and *B. truncatus* snails were exposed to a light source to induce shedding of live *S. mansoni* and *S. haematobium* cercariae. Avian schistosome cercariae, of the putative species *Austrobilharzia penneri*, were obtained from laboratory of Prof. W.P. Sousa of the University of California at Berkeley. Cercariae were preserved in 95% ethanol in 1.5 mL microcentrifuge tubes.

### Light microscopy

Cercariae from crushed snails or shed in water were isolated using a 3 mm diameter inoculation ring (Fisher Scientific, Pittsburgh, USA). Cercariae trapped within the inoculation ring were counted under a compound microscope, followed by an inspection under a dissecting microscope for any cercariae adhered to the ring. The quantified cercariae were transferred to 300 µL of 95% ethanol in a 1.5 mL microcentrifuge tube. The procedure was repeated until a specified number of cercariae were obtained in one sample. All samples were stored at −20°C until DNA was extracted from the cercariae.

### Extraction of genomic DNA from schistosomes

Samples of quantified cercariae preserved in 95% ethanol were centrifuged at 1,200 rpm for 10 minutes, after which the supernatant was discarded. The samples were air dried before genomic DNA (gDNA) extraction was performed using a DNeasy Tissue Kit (Qiagen, CA, USA) following the manufacturer's instruction. Schistosome gDNA was eluted with 100 µL of AE buffer from the DNeasy column, and stored at −20°C until analyzed by quantitative PCR (qPCR).

### Design of primers and probe

Three primer sets were designed targeting the *SjR2* retrotransposon [11], the putative deoxyribodipyrimidine photo-lyase [18], and the *Merlin* transposon [19] (Table 1). The three primer sets were used in conventional PCR and SYBR Green qPCR (Applied Biosystems, Foster City, CA, USA), a reagent mix containing a specific dye for quantification, for the detection of *S. japonicum* cercarial DNA. The primer set designed from the putative deoxyribodipyrimidine photo-lyase (PL) sequence provided the highest correlation between counted concentration of cercariae and real-time PCR estimation, and was thus selected for further development using the TaqMan system.

**Table 1:** Oligonucleotide primers and probe sequences for real-time PCR.

Primers and probe	Sequences (5′→3′)	Target	Function	GenBank accession no.
Mer-F	ATCAAAGGATACTGGTCACACCTAAA	*Merlin*	DNA transposon	BU779421
Mer-R	TGGCCCCATATCATGTTGTTT			
PL-F	GCCTTCTTGTTTGCTCAACGT	SJCHGC08270 protein mRNA	putative DNA photo-lyase	AY812553
PL-R	CCGCTTGATATTTTGGAACGA			
PL-PR	FAM-TAGCGTTAAAATTTAAAGTCCCTCTCCATGTTTGTTTCT–TAMARA			
SjR2-F	CCCTATAACAAGACCGTGCGTAA	SjR2	retrotransposon	AF412220
SjR2-R	CTTGGAGCACGATCGCAAA			

Primers and probe were designed using Primer Express software version 2.0 (Applied Biosystems). The probe was designed with 5′ terminal reporter dye FAM (6-carboxyfluorescein) and 3′ terminal quencher dye TAMRA. The specificity of primers and probe were tested using a BLAST search against the Genbank database to assess potential cross hybridization with other schistosome species and other non-target organisms.

### Real-time PCR conditions

Real-time quantitative PCR was performed in MicroAmp optical 96-well reaction plates using StepOnePlus Real-Time PCR Systems (v. 2.0, Applied Biosystems). Primer concentrations were optimized by testing various annealing temperatures (57°C, 59°C, 60°C, 61°C, 63°C), and various concentrations of magnesium chloride (0 nM, 250 nM, 500 nM, 750 nM). All real-time PCR assays were run in a total reaction volume of 25 µL comprised 1×TaqMan Universal PCR Master Mix with AmpErase (Applied Biosystems), 500 nM of both forward and reverse primers (Integrated DNA Technologies, Coralville, IA, USA), 240 nM TaqMan probe (Applied Biosystems) and 2 µL of target DNA solution.

The real-time PCR cycling parameters were set as follows: initial 2 minutes at 50°C for optimal UNG enzyme activity followed by 95°C for 10 minutes to activate AmpliTaq gold enzyme, then 40 cycles of 15 seconds at 95°C and 1 minute at 60°C. Standards were prepared from ten-fold serial dilutions of extracted DNA solution from samples containing 500 microscopy counted *S. japonicum* cercariae. Negative controls without template were run on each plate using nuclease-free water. Species specificity of the assay was tested against two other human schistosomes, *S. mansoni* and *S. haematobium*; and one avian schistosome, *A. penneri*. Ten to twenty cercariae of the two schistosome species were counted, gDNA was extracted (as above) and was subsequently assayed. All standards, samples and controls were carried out in triplicate. Unknown sample concentrations were calculated from a standard curve of samples of known concentration run on the same plate. A sample was considered positive when more than one out of three reactions were positive. Some real-time PCR products were assayed by 2% agarose gel electrophoresis stained by ethidium bromide to confirm the expected size of the amplicons.

### Filtration of spiked water samples and DNA extraction

Samples spiked with live cercariae were prepared for filtration using three different water sources: first, deionized water, second, phosphate buffered saline (1×), and third using water taken directly from the Strawberry Creek Canyon on the campus of University of California, Berkeley. This third water source was included to represent environmental water and mimics natural water sources in schistosomiasis endemic sites. Each one liter water sample was spiked with a specific number of *S. japonicum* cercariae counted using light microscopy. Subsequently, samples were filtered through a Whatman Nuclepore membrane filter (8.0 µm pore size, 47 mm diameter, Tracked-Etched Nuclepore membrane; Fisher Scientific, Pittsburgh, USA). Each sample flask was rinsed with deionized water five times and the rinse water was added to the filter. The filter membrane was folded four times using sterilized forceps, transferred to a 2.0 mL microcentrifuge tube, and stored at −20°C until DNA extraction. Several spiked water samples (n = 4) were treated with bead beating as part of DNA extraction. 360 µL Buffer ATL (DNeasy Tissue Kit; Qiagen, CA, USA), and beads (0.5 g, 0.1 mm diameter; Biospec Products, Bartlesville, USA) were added to the folded filter membrane. Contents were homogenized for 1 minute at 500 g in a mini-bead beater (Biospec Products, Bartlesville, USA). Samples treated with bead beating were later compared with samples without bead beating.

DNA was extracted using a modified DNeasy Tissue Kit protocol (Qiagen, CA, USA). Samples were lysed in twice the suggested volume of buffer ATL and proteinase K to allow for sufficient contact with the filter; the lysates were incubated for two hours with vortexing at 15 minute intervals to ensure contents were homogenized. Samples were run through a single column twice with centrifugation. Purified DNA was eluted once with 100 µL buffer AE in the final step. Strawberry Creek Canyon stream water without spiked cercariae was prepared and assayed as a negative control in parallel with the spiked samples. DNA extracted from samples spiked in the stream water was processed by filtration and diluted ten-fold in nuclease-free water to test for the potential presence of real-time PCR inhibitors.

## Results

### Real-time PCR specificity and optimization

A BLAST search of the chosen primer and probe sequence resulted in a single hit of the target sequence in *S. japonicum*, suggesting specificity of the target. Moreover, no amplification was observed from gDNA extracted from *S. mansoni*, *S. haematobium*, *A. penneri* cercariae, nor from DNA extracted from filtered stream water. Visualization of real-time PCR products on agarose gel confirmed the amplification of the specific template of expected size (85 bp; data not shown).

Results of real-time PCR optimization using three concentrations of magnesium chloride, five annealing temperatures, and two primer concentrations showed similar results for 500 nM and 900 nM primer concentrations, and an optimal annealing temperature of 60°C (Table 2). Additional magnesium chloride reduced sensitivity (data not shown). Reactions using optimized cycling parameters generated an average amplification efficiency of 91%. Standard curves generated from 10-fold serial dilutions of gDNA extracted from 500 cercariae were linear over 3 orders of magnitude. The average slope of standard curves was −3.58, with a standard deviation of 0.27. The real-time PCR detection limit was DNA equivalent to half of one cercaria, with a mean Ct (cycle threshold) value of 36.7.

**Table 2:** Real-time PCR optimization varying annealing temperature and primer concentration.

	Primer Concentration	Temperature				
		57°C	59°C	60°C	61°C	63°C
**Ct value**	500 nM	27.54	27.31	26.48	26.73	29.12
	900 nM	27.4	26.55	26.47	26.5	28.45

### Application of real-time PCR method to laboratory samples

**Figure 1:**
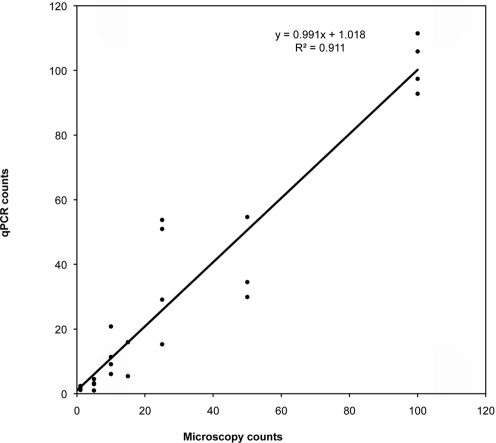
Correlation between real-time PCR and microscopy counts of cercariae recovered from *Oncomelania hupensis*. A total of 25 samples were performed and compared at different cercarial concentrations. Real-time PCR reactions were run in triplicate.

The optimized real-time PCR method was applied to 25 independently prepared laboratory samples of various cercarial concentrations, varying from 1 to 100 cercariae, all of which were positively detected. Moreover, a statistically significant, positive linear correlation was observed between the real-time PCR estimated number and the light microscopy counts when analyzed by linear regression (Figure 1; R^2^ = 0.912, slope = 0.992, 95% CI = 0.859–1.125). Mean real-time PCR readings generally agreed with the cercarial numbers as determined by light microscopy, although a range of values was observed when replicates of the same concentration of cercariae were analyzed (Table 3). The results were reproducible when the real-time PCR was carried out on the same gDNA samples on different days (data not shown).

**Table 3:** Real-time PCR detection of samples counted with microscopy.

No. of cercariae	No. of replicates	Real-time PCR count (mean±SD)	Range
100	4	101.9±8.4	92.8–111.5
50	3	39.7±13.2	29.9–54.7
25	4	37.3±18.3	15.3–54.7
15	2	10.7±7.5	5.4–15.9
10	4	11.8±6.3	6.1–20.0
5	4	2.9±1.5	1.0–4.5
1	4	1.8±0.5	1.1–2.4

### Real-time PCR of filtered samples

A comparison of filtered environmental samples treated by bead beating (n = 4) and samples with direct DNA extraction from membranes (n = 5) showed that bead beating significantly lowered the quantitative estimate of cercarial concentration in samples when analyzed by real-time PCR (data not shown). All samples were thus processed by direct DNA extraction from membranes without bead beating.

All spiked deionized water and PBS samples resulted in positive detection (100%, n = 16) (Table 4). Cercarial concentration as low as one cercaria per liter of stream water was tested in duplicate with filtration, with one of the replicates detected. Overall, positive results were obtained in 93% of all filtered samples spiked with cercariae (n = 32) and analyzed by real-time PCR (Table 4). Samples spiked with higher numbers of cercariae (>10 cercariae) had a higher probability of being detected after filtration. The findings with spiked stream water suggested the presence of environmental inhibitors. Undetectable amplification signals were observed in undiluted samples, but subsequent dilutions produced detectable amplification (data not shown). DNA dilutions of 2-fold, 10-fold and 100-fold in stream water samples reduced inhibition effects. A comparison across different concentrations of spiked cercariae suggested optimal detection at 10-fold dilution. Subsequently, samples of spiked stream water were analyzed at this dilution.

**Table 4:** Recovery of cercariae in filtration.

No. of cercariae spiked	Detection in matrix (positive/total)		
	Stream water	PBS	DI water
100	2/2	ND	2/2
50	5/5	3/3	3/3
25	2/2	ND	3/3
10	4/5	3/3	2/2
1	1/2	ND	ND

ND: not determined; PBS: 1×phosphate buffer solution; DI water: deionized water.

In general, high variability was observed in spiked samples with filtration when compared to direct DNA extraction of cercariae without filtration (Table 5). Visualization of real-time PCR products on ethidium bromide stained agarose gels suggested that there were no other amplifications (data not shown). Repeated assays of negative controls did not amplify, which was further confirmed by analysis of the real-time PCR products on agarose gel.

**Table 5:** Real-time PCR quantification of spiked stream water samples after filtration.

No. of cercariae spiked	No. of replicates	Cercariae recovered (mean±SD)
100	2	163.7±44.3
50	5	52.3±34.9
25	2	22.6±24.6
10	3	17.5±11.4
1	1	2.3

## Discussion

In order to overcome the cost and time limitations of current methods for quantification of *S. japonicum* cercariae in natural water, we have developed and evaluated a real-time PCR assay. Multiple target sequences were assessed, and a TaqMan real-time PCR assay was designed and applied to cercariae recovered from *O. hupensis* snails, and from spiked water samples. Results demonstrated the specificity of the target sequence, a putative deoxyribodipyrimidine photo-lyase, which amplified *S. japonicum*, but not other closely related human schistosomes, nor organisms present in environmental water samples drawn from a non-endemic area. The authors acknowledge the limitations of the sensitivity assessments available in this study, including environmental samples drawn from a non-endemic area and BLAST searches of sequence databases where trematode sequences are often poorly represented. Other PCR-based molecular assays developed to detect human schistosomes were generally species specific when tested against related trematodes [11], [12], [20]–[24]. However, as with previous assays, the authors of the current work remain cautious, recommending additional field testing for cross-hybridization with other schistosome species to confirm specificity of the assay presented here [25].

For a robust and quantitative assay, we selected a target sequence with a fixed number of copies from a highly conserved region in the *S. japonicum* genome. The target sequence encodes a putative deoxyribodipyrimidine photo-lyase which is expressed only in living cercariae [18]. The selective expression of this protein could be extremely valuable for the future development of a reverse transcriptase PCR method to detect the level of mRNA, thus allowing for the quantification of viable cercariae. Taking advantage of the target developed in this project, future efforts on a reverse transcriptase PCR method could offer the added benefit of a quantitative technique that accounts for viability, which would be especially valuable for improving risk assessments [26]. Conventional PCR methods developed to detect cercariae have usually targeted highly repeated sequences to enhance the assay's sensitivity, demonstrating in several cases the capability to detect a single cercaria [11],[12],[23]. In this study, we demonstrated sensitive detection using a non-abundant target sequence, capable of detecting one cercaria in replicated samples, as well as the DNA equivalent of half a cercaria.

Quantification is a major step forward in *S. japonicum* detection, since current methods using mouse bioassays are minimally quantitative, and prior PCR methods have allowed for qualitative detection only. The assay presented here showed a significant association between Ct values and the concentration of cercarial DNA, both from standards derived by serial dilution, and from individual counted samples. This strong association suggests the standards provided a reliable quantitative estimation of individual samples, derived from relative Ct values. The variance in real-time PCR quantification within replicates of the same cercarial number could be due in part to error in light microscopy quantification. Larger errors in high concentration samples could be expected because of the possibility of multiple miscounts in a single concentrated sample.

In the spiked water samples processed with a filtration step, high variability was observed when compared with samples processed without filtration. Samples spiked with ≤10 cercariae had a lower probability of being detected (83% compared to samples >10 cercariae, n = 12). While DNA losses in the filtration and DNA extraction steps are expected, a large variance was observed in real-time PCR estimates, rather than a simple underestimation. Thus, the recovery efficiency in the filtration process could not be determined. Subsequent testing may include multiple replicates of samples with a larger range to determine the sampling variability. The variance observed could be attributed to inhibitors present in environmental water samples; indeed, inhibition effects were observed in the stream water. It has been reported that at different concentrations, the same compound can act as both attenuator and facilitator in the same system [27]. In our environmental samples, the presence of excessive amounts of non-target DNA and humic compounds might affect several reaction steps in real-time PCR. To reduce the problem of inhibition, studies on other parasites in environmental samples have used bovine serum albumin (BSA) [28]–[31]. Further refinement of the assay presented here might explore BSA, among other approaches, to reduce inhibitory effects.

Real-time PCR techniques have been used to detect eggs in stool samples for several schistosome species [14]–[16], providing a diagnostic tool for clinical applications. Here, we have demonstrated the promising performance of a similar technique applied to the quantification of *S. japonicum* cercariae in natural water samples, useful for surveillance, risk assessments and intervention planning. To adapt the method to field sampling, the assay should be assessed in parallel with mouse bioassays which, despite their limitations, remain the gold standard detection technique in endemic areas [11]. Such a field trial could reveal potential limitations of the qPCR assay in comparison to the sentinel animal assay. For example, the sentinel mouse assay selectively detects viable cercariae, and could theoretically take advantage of cercarial host-seeking behavior to detect very low concentrations of cercariae, or cercariae in highly turbid environments. Indeed, given the typical turbidity of water in endemic environments in western China, sedimentation prior to filtration may be required to reduce loading of filter membranes. Moreover, as cercarial concentration may be lower than 1 cercaria per liter of water in endemic areas, an effective water sampling technique capable of skimming surface water for cercariae may be necessary in areas of low cercarial concentration, taking advantage of the surface seeking behavior of cercariae [32]. Additional sampling strategies such as filtration or centrifugation may be necessary in future development of the method application. The assay presented here can serve as the foundation for a rapid technique for the analysis of environmental samples, and it has the potential to improve the temporal and spatial resolution of cercarial density measurements.
